# Type 2 Dendritic Cells Orchestrate a Local Immune Circuit to Confer Antimetastatic Immunity

**DOI:** 10.4049/jimmunol.2200697

**Published:** 2023-03-03

**Authors:** Orr-El Weizman, Sophia Luyten, Irina Krykbaeva, Eric Song, Tianyang Mao, Marcus Bosenberg, Akiko Iwasaki

**Affiliations:** * Department of Immunobiology, Yale University School of Medicine, New Haven, CT; † Department of Dermatology, Yale University School of Medicine, New Haven, CT; ‡ Department of Pathology, Yale University School of Medicine, New Haven, CT; § Howard Hughes Medical Institute, Chevy Chase, MD

## Abstract

The progression of transformed primary tumors to metastatic colonization is a lethal determinant of disease outcome. Although circulating adaptive and innate lymphocyte effector responses are required for effective antimetastatic immunity, whether tissue-resident immune circuits confer initial immunity at sites of metastatic dissemination remains ill defined. Here we examine the nature of local immune cell responses during early metastatic seeding in the lung using intracardiac injection to mimic monodispersed metastatic spread. Using syngeneic murine melanoma and colon cancer models, we demonstrate that lung-resident conventional type 2 dendritic cells (DC2) orchestrate a local immune circuit to confer host antimetastatic immunity. Tissue-specific ablation of lung DC2, and not peripheral DC populations, led to increased metastatic burden in the presence of an intact T cell and NK cell compartment. We demonstrate that DC nucleic acid sensing and transcription factors IRF3 and IRF7 signaling are required for early metastatic control and that DC2 serve as a robust source of proinflammatory cytokines in the lung. Critically, DC2 direct the local production of IFN-γ by lung-resident NK cells, which limits the initial metastatic burden. Collectively, our results highlight, to our knowledge, a novel DC2–NK cell axis that colocalizes around pioneering metastatic cells to orchestrate an early innate immune response program to limit initial metastatic burden in the lung.

## Key Points

- DC2 limit initial metastatic burden in the lung.
- In response to tumor-derived nucleic acids, DC2 produce host-required IL-12p40.
- Local DC2 and NK cells coordinate initial metastatic control.

## Visual Abstract

**Figure:**
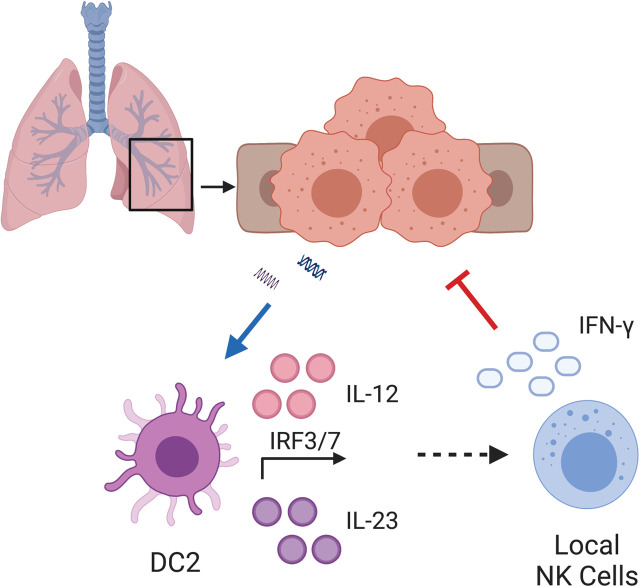

## Introduction

Tumor metastasis requires a multistep process of dissemination of cancer cells to seed anatomically distant sites and the subsequent successful adaptation and colonization to form metastatic lesions (1, 2). Distinct immune responses play competing, supportive, and protective roles in shaping metastatic progression. Multiple lines of evidence demonstrate the supportive nature of circulating monocytes and tissue-resident macrophages in preparing the premetastatic niche and limiting late-stage, antimetastatic adaptive responses (3, 4). Conversely, circulating tumor cells from a wide array of primary tumors are a target of circulating NK cell–mediated killing in the periphery (5, 6). Indeed, more primary tumor-infiltrating or circulating NK cells inversely correlates with the clinical presentation of metastatic disease, indicating a unique role for NK cell immune surveillance of metastases (5, 7). Following the seeding of distal tissues, particles derived from pioneering tumor cells are phagocytosed and ingested sequentially by multiple waves of myeloid cells (8). Although most phagocytosis is performed by metastasis-supporting myeloid cells, ingested particles by type 1 conventional dendritic cells (DC1) in the lung are required to initiate antimetastatic CD8^+^ T cell responses in the draining lymph node (8). However, prior to the initiation and recruitment of adaptive responses, little is known about whether the local immune system can limit the initial metastatic burden to confer immediate host protection at sites of metastatic seeding.

It is increasingly apparent that conventional dendritic cells (DC) are critical regulators of complex immune response circuits that allow the targeting and elimination of a wide variety of potential threats, from pathogenic challenges to cancerous transformations (9, 10). This is in part due to the superior ability of DC as both sensors of local perturbations and translators of activating signals to specific effector cell lineages (9, 11). DC can be divided into type 1 and type 2 DC (DC1 and DC2, respectively) delineated by the lineage-defining transcription factors IFN regulatory factor 8 (IRF8) and IRF4 and the surface markers XCR1 and CD172a, respectively (10, 12, 13). Due to unique cytokine production profiles and specialized Ag presentation capabilities, the division of labor between DC1 and DC2 is well documented in directing elaborate adaptive CD8^+^ and CD4^+^ T cell responses, respectively, for both tumor and pathogen challenge (14–20). Recent evidence highlights a nonredundant role for DC1 and DC2 in orchestrating innate lymphocyte cell (ILC) responses to limit viral, bacterial, and fungal burdens prior to the recruitment of the circulating adaptive response (9, 21–23). It remains unclear whether DC play a similar role as tissue sentinels orchestrating immune efforts to limit initial metastatic seeding.

In this study, we report that local immune cells interact with pioneering metastatic cells and actively limit the initial metastatic burden in the lung. Specifically, we demonstrate a novel DC2-controlled circuit that directs local NK cells to confer initial host-derived antimetastatic immunity in an IFN-γ-dependent manner, whereas other myeloid populations and adaptive lymphocytes appear dispensable. Among DC populations, lung DC2 served as the sole producer of proinflammatory cytokines IL-12p40 and IL-23p19. Initial antimetastatic control required DC-intrinsic IRF3 and IRF7 signaling downstream of nucleic acid sensors. Ablation of DC2, but not DC1, abrogated the local production of IFN-γ by NK cells in the lung but not the periphery, resulting in increased metastatic burden. Thus, our study provides insights into the essential role of DC2 in orchestrating tissue responses prior to recruited circulating responses in the host, leading to antimetastatic immunity.

## Materials and Methods

### Experimental model and subject details

#### Mice

Mice were bred at Yale University in accordance with the guidelines of the institutional animal care and use committee. The following mouse strains were used in this study: C57BL/6J (CD45.2, wild type [WT]), C57BL/6N, CD11b^DTR^, CD11c^DTR^, *Ccr2*^−/−^, CD169^DTR^, Mgl2^DTR^, DC^DTR^ (Zbtb46^DTR^), XCR1^DTR^, *Ticam1*^−/−^ (TRIF^−/−^), *Tlr7*^−/−^(TLR7^−/−^), *Mb21d1*^−/−^ (cGas^−/−^), MAVs^−/−^ (from Dr. Z. Chen, University of Texas Southwestern, Dallas, TX), cGas^−/−^xMavs^−/−^, IRF3^−/−^xIRF7^−/−^ (IRF3/7^−/−^; from T. Taniguchi, University of Tokyo, Tokyo, Japan), *Rag2*^−/−^, *Tcra*^−/−^, and *Tcrd*^−/−^. All mice were purchased from The Jackson Laboratory unless otherwise specified. Experiments were conducted using age- and sex-matched mice in accordance with approved institutional protocols.

#### Cancer cell lines

YUMMER1.7 stably expressing GFP and luciferase (YMR) was generating using retroviral transduction (Yale, M. Bosenberg). The YMR cell line was cultured in DMEM/F-12 (Thermo Fisher, 11320-033), including l-glutamine and 2.438 g/L sodium bicarbonate, and supplemented with 1× nonessential amino acids (Thermo Fisher, 11140050), 10% FBS (Thermo Fisher, 16140-071), and 1× penicillin and streptomycin (Thermo Fisher, 15140-122). MC38 stably expressing luciferase (MC38) was generated using retroviral transduction (Yale Center for Precision Cancer Modeling, V. Muthusamy). The MC38 cell line was cultured in DMEM (Thermo Fisher, 11965092), including l-glutamine, and supplemented with 1× nonessential amino acids (Thermo Fisher, 11140050), 10% FBS (Thermo Fisher, 16140-071), and 1× penicillin and streptomycin (Thermo Fisher, 15140-122). MC38 were kept under blasticidin (10 μg/ml; InvivoGen) and geneticin (100 μg/ml, InvivoGen) selection. All cells were cultured at 37°C, 5% CO_2_, and kept at low passage (approximately three to five passages) and showed negative test results for mycoplasma contamination.

### Method details

#### Metastatic inoculation and burden measurement

Mice were anesthetized using ketamine and xylazine, and the ventral thoracic region was shaved and disinfected. A 1-ml syringe with a 26-gauge needle was used to deliver 100 μl of either 5 × 10^4^ or 5 × 10^5^ YMR cells or 1 × 10^5^ MC38 in PBS intracardially (i.c.) to the left ventricle or 1 × 10^5^ YMR cells i.v. All samples were filtered in 40-μm mesh filter prior to injection to ensure single-cell suspension. Control injected mice received i.c. injection with PBS alone.

Direct measurement of whole-tissue metastatic burden was performed on anesthetized mice by administering 100 μl of 15 μg/ml of D-Luciferin (PerkinElmer) i.v. and euthanized 2 min later. Following dissection of the lung and spleen, samples were imaged for bioluminescence (IVIS, PerkinElmer) at 1-min exposure or for GFP microclusters (Stereoscope, Leica 205). Day 3 metastatic burden is shown as the normalization of average radiance to day 3 metastatic tumor–bearing WT mice. For longitudinal metastatic studies, mice were anesthetized and injected i.v. with 100 μl of 15 μg/ml of D-Luciferin. Following 2-min incubation, the ventral region of mice was imaged at 1-min exposure. Imaging was continually performed biweekly using the same instrument and exposure parameters until terminal endpoint. Longitudinal metastatic burden is shown as the normalization of average radiance to initial bioluminescent signal measured following i.c. injection. Bioluminescent imaging was performed using the IVIS spectrum imager (PerkinElmer), and average radiance was measured using the ROI tool in Living Image 4.4 software (PerkinElmer).

#### Mouse techniques

For bone marrow chimera (BMC) experiments, 5–6-wk-old mice were irradiated with two doses of 475 rad and transplanted with ∼10^6^ bone marrow cells i.v. In mixed BMC experiments, IRF3/7^−/−^ bone marrow was mixed 1:1 with either WT (B6J, 45.2) or DC^DTR^. All BMC mice were treated with Sulfatrim in drinking water for 2 wk and then maintained on regular water for another 6 wk or longer before being used in experiments.

For diphtheria toxin receptor (DTR) ablation studies, WT and indicated DTR mice received injection twice intratracheally (i.t.) with 100 ng diphtheria toxin (DT) in 20 μl of sterile PBS or i.p. with 200 ng DT in 100 μl of sterile PBS (150, List Biological Lab) on 1 d before and after metastatic inoculation. Cell of interest depletion was confirmed by flow cytometry.

For T cell and NK cell depletion and IFN-γ and IL-12p40 neutralization studies, Abs against CD8a (YTS169.4, Bio X Cell), CD4 (GK1.5, Bio X Cell), NK1.1 (PK136, Bio X Cell), IFN-γ (H22, BioLegend), and IL-12p40 (C17.8, Bio X Cell) were used. Cell depletion Abs were administered by i.p. injection starting on days 3 and 1 prior to metastatic i.c. injection and on day 1.5 after injection. IFN-γ and IL-12p40 neutralization Ab was administered i.p. 6 h prior to metastatic injection and continued every day for the duration of the experiment. All mice were dosed ∼4 mg/kg (100 μg/mouse) in 100 μl, except for IL-12p40 treatment, which was dosed 20 mg/kg (500 μg/mouse) in 500 μl. Lymphocyte depletions were confirmed in PBLs by flow cytometry with the following Abs: CD8a (53-6.7, BioLegend), CD4 (RM4-5, BioLegend), and NKp46 (29A1.4, BioLegend).

Intravascular labeling of lymphocytes of experimental mice was performed by injecting (i.v.) 2.5 μg of fluorophore-conjugated CD45 (30-F11) or CD45.2 (104), and mice were euthanized 3 min later.

#### Microscopy

Prior to tissue harvesting, mice were perfused with 10 ml of PBS followed by 10 ml of BD Cytofix/Cytoperm Buffer (BD Pharmingen; diluted 4× in PBS). Harvested lung was fixed in diluted BD Cytofix/Cytoperm for 24 h. Fixed tissues were dehydrated in 30% sucrose prior to embedding with optimal cutting temperature medium (Tissue-Tek) and frozen at −80°C. Frozen sections of 20–22-μm thicknesses were cut and left to dry at ambient temperature. Sections were blocked with 5% BSA, 0.05% Triton X-100, and PBS (blocking buffer) and stained overnight at 4°C with the following primary Abs: CD45-biotin (30-F11, BioLegend, 1:200), CD11c–Alexa Fluor 647 (N418, BioLegend, 1:200), and NK1.1-biotin (PK136, BioLegend, 1:200). Sections were then washed twice at room temperature with blocking buffer before incubation with a fluorochrome-conjugated secondary Ab (streptavidin Cy-3, Jackson ImmunoResearch, 1:500) at room temperature for 1.5 h. Slides were washed twice with blocking buffer prior to incubation with DAPI and washed once with deionized water. Sections were mounted with SlowFade Diamond Antifade (Fisher Scientific). Stained tissues were then analyzed by fluorescence microscopy (BX51, Olympus) or confocal microscopy (TCS SP8, Leica). Three-dimensional surface rendering and closest distance calculations were completed using Imaris 8 software (Oxford Instruments).

#### Isolation of lymphocytes and myeloid cells

Spleens and mesenteric lymph nodes were dissociated using glass slides and filtered through a 70-µm strainer. To isolate cells from the lung, the tissue was physically dissociated using gentleMACS Dissociators (Miltenyi Biotec) and incubated for 40 min in digest solution (1 mg/ml type D collagenase, Roche) and 10 U/ml DNase I (Roche) and in RPMI supplemented with 5% FCS, 1% l-glutamine, 1% penicillin-streptomycin, and 10 mM HEPES. The resulting dissociated tissue was passed through 70-µm strainers, then centrifuged, and lymphocytes were removed from the supernatant. RBCs were lysed using ammonium-chloride-potassium lysis buffer.

#### Flow cytometry and analysis

Flow cytometry and cell sorting were performed on the Attune NXT (Thermo Fisher) and FACSAria II (BD Biosciences), respectively, and data were analyzed with FlowJo software (BD Biosciences). Cell surface staining of single-cell suspensions from various organs was performed using fluorophore-conjugated Abs (BD Biosciences, Thermo Fisher, and BioLegend). Flow cytometry Abs used in analysis can be found below. Intracellular staining was performed by fixing and permeabilizing with the eBioscience Foxp3/Transcription Factor Staining Set (Thermo Fisher) for staining intranuclear proteins. Intracellular staining for IFN-γ was performed after 4-h incubation of single-cell lung suspensions in RPMI containing 10% FBS with brefeldin A (10 µg/ml, Sigma-Aldrich). Intracellular staining for ex vivo IL-12p40/70 and IL-23p19 of DCs from metastatic tumor–bearing lungs was performed after 6-h incubation of single-cell lung suspension in RPMI containing 10% FBS with cell stimulation and protein transport inhibitor mixture (500×, eBioscience). Intracellular staining for in vitro IL-12p40 of naive sort-purified lung DCs was performed after 12-h incubation with YMR media alone or treated with DNase and RNase with protein transport inhibitor mixture (500×, eBioscience).

Cell surface and intracellular staining was performed using the following fluorophore-conjugated Abs: NK1.1 (PK136), CD11b (M1/70), CD19 (ID3), CD49b/DX5 (DX5), KLRG1 (2F1), NKp46 (29A1.4), CD45 (30-F11), CD45.2 (104), CD8a (53-6.7), CD4 (GK1.5), TCRβ (H57-597), Eomesodermin (Dan11mag), CD3e (17A2), CD90.2 (30-H12), CD200r1 (OX-110), CD11c (N418), IFN-γ (XMG1.2), MHC class II (M5/114.14.2), CD64 (X54-5/7.1), XCR1 (ZET), CD172a (P84), CD169 (MA5-16508), CD301b (11A10-B7), FOXP3 (FJK-16s), IL-12p40/70 (C15.6), and IL-23p19 (fc23cpg).

#### Human data analyses

The gene expression data from the MET500 cohort were downloaded through UCSC Xena (https://xenabrowser.net/datapages/?cohort=MET500%20(expression%20centric)). For correlation analysis, raw gene expression was first converted to fragments per kilobase per million mapped reads, followed by log_2_(*x* + 0.001) transformation. A DC2 signature was defined as the average expression of *IRF4*, *CD1A*, *CD1C*, and *CLEC10A*; an NK cell signature was defined as the average expression of *NCR1*, *KLRD1*, *KLRF1*, *KLRC3*, and *KIR2DL4*; and a monocyte/macrophage signature was defined as the average expression of *FCN1*, *VCAN*, *CD14*, *CD68*, *FCGR1A*, *S100A8*, *S100A9*, and *S100A12*. All signatures were validated against a publicly available single-cell atlas of immune cells (24–26). Pearson’s correlation coefficients and linear regression significance were then calculated to assess the association between the indicated comparison in metastatic tumor samples. Carcinomas of unknown primary were excluded from the analysis.

## Results

### Local immune cells are recruited to interact with metastatic cells in the lung

**FIGURE 1.**
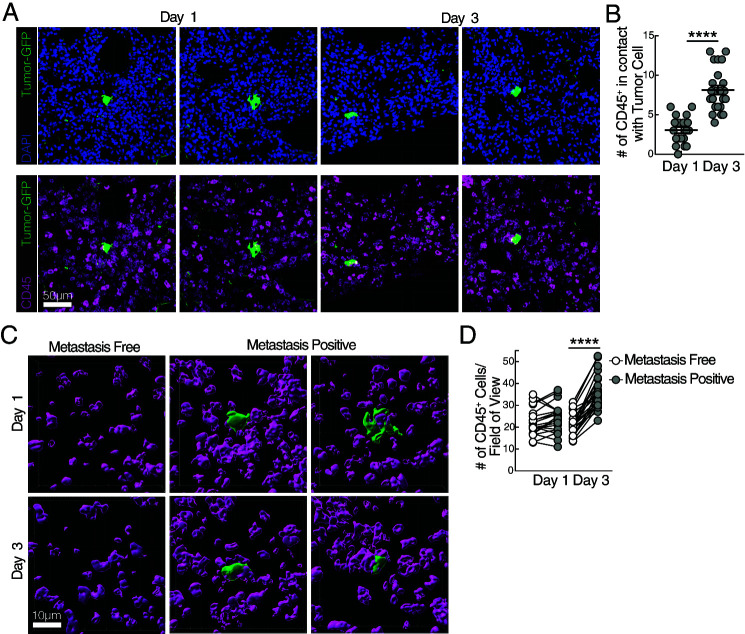
CD45^+^ immune cells are locally recruited to interact with pioneering metastatic cells in the lung. WT mice received an injection with 5 × 10^5^ YUMMER1.7 cells coexpressing GFP and luciferase (YMR) i.c. (**A**) Representative confocal image of frozen lung section depicting GFP^+^ YMR cell (GFP, green; top) colocalizing with CD45-stained cells (magenta; bottom) costained with DAPI (blue) at days 1 and 3 p.i. (**B**) Quantification of the number of CD45^+^ cells in direct contact with YMR cell on day 1 and 3 p.i. in field of view. (**C**) Imaris rendering of three-dimensional surface interactions between CD45^+^ cells and GFP^+^ YMR cell on days 1 and 3 p.i. (**D**) Paired quantification of the number of CD45^+^ cells in metastasis-free section compared with metastasis-positive section present in the same field of view. (See Supplemental Fig. 1D for additional images.) Data are representative of two independent experiments with *n* = 3 mice per group. (B, D) Quantification data are from 120 metastasis-free sections and 30 metastasis-positive sections per time point. Sample is compared using (B) paired Student *t* test and (D) unpaired Student *t* test, and data are presented as mean ± SEM. ****p < 0.0001.

We initially sought to determine the nature of the immune landscape that interacts with initial metastatic cells in distal sites. Thus, we employed an experimental metastatic model using syngeneic mouse melanoma YUMMER1.7 tumors expressing GFP and luciferase (YMR) injected i.c. into the arterial circulation of WT mice, which allowed systemic monodispersing of metastatic cells (27, 28). Monitoring by direct bioluminescence imaging of dissected tissues revealed that only the lung and the spleen had detectable luciferin signals as early as day 1 postinjection (p.i.) compared with naive controls (Supplemental Fig. 1A, 1B). However, in the spleen, this was followed by rapid reduction in luciferin signal that became undetectable (Supplemental Fig. 1A), suggesting that the spleen could not survive early metastatic outgrowth in this model. Although there was an initial reduction of luciferin signal in the lung from day 1 to day 2 p.i., we observed stable outgrowth in the luciferin signal from day 2 onward in the lung (Supplemental Fig. 1B). This observed kinetics is consistent with previous work establishing the initial increased frequency of fragmented tumor particles, a target of immediate immune-mediated removal, relative to surviving tumor cells upon extravasation of circulating tumor into distal sites (8). Using immunofluorescence microscopy of lung sections from metastatic tumor–bearing mice, we identified the presence of monodispersed, single-nucleated GFP^+^ YMR cells on day 1 and day 3 p.i. that increased in number and cluster size by day 7 p.i. (Fig. 1A, Supplemental Fig. 1C), confirming the presence of surviving metastatic cells in the lung.

Staining of the lungs of mice bearing metastatic cells revealed that CD45^+^ cells colocalized around GFP^+^ YMR cells on days 1, 3, and 7 after i.c. injection (Fig. 1A, Supplemental Fig. 1C) with more CD45^+^ cells in physical contact with a YMR cell on day 3 p.i. than on day 1 p.i. (Fig. 1B). Notably, paired comparison of metastasis-free (no detectable GFP^+^ cells) and positive sections (at least one GFP^+^ cell) within the same lung from metastasis-bearing mice (Fig. 1C, Supplemental Fig. 1D) demonstrated a significant increase in CD45^+^ cells in relation to metastatic tumor cells on day 3 p.i. compared with day 1 p.i. (Fig. 1D). We observed no differences in the total absolute cell number of extravascular myeloid and lymphocyte population in the lungs of day 3 metastasis-bearing mice compared with naive control mice as assessed by flow cytometry (Supplemental Fig. 1E, 1F), suggesting that neither local proliferation nor recruitment of peripheral immune cells occurs during this time frame. Together, these results suggest that immune cells in the lung are recruited in a local manner to interact with pioneering, individual metastatic cells.

### DC2 are required to confer early metastatic control at initial sites of tumor seeding

**FIGURE 2.**
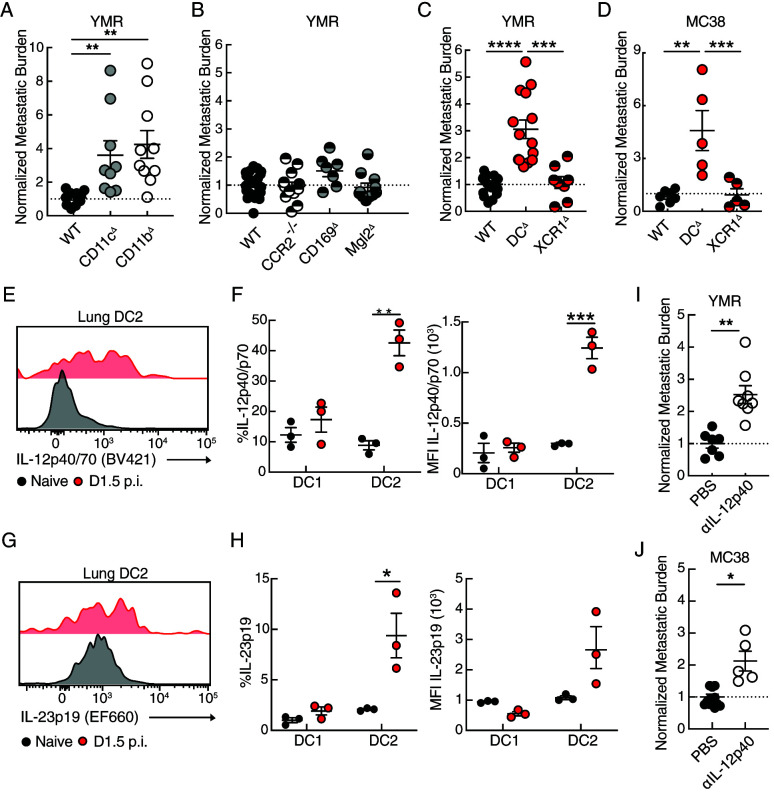
Lung DC2 confer early host antimetastatic immunity. (**A**–**C**) Mice received injection with 0.5 × 10^5^ YMR cells i.c., and whole-lung tissue was analyzed on day 3 p.i. All mice were treated with DT 1 d before and after metastatic inoculation. (A–C) Quantification of metastatic burden in the lung of (A) CD11b^DTR^ BMC, CD11c^DTR^ BMC, (B) *Ccr2*^−/−^, CD169^DTR^, Mgl2^DTR^, (C) DC^DTR^ BMC, XCR1^DTR^, and WT mice measured by average radiance. Data are shown as the normalization of average radiance to day 3 in metastasis-bearing WT mice. (**D**) Quantification of metastatic burden in the lung of DC^DTR^ BMC, XCR1^DTR^, and WT mice injected with 1 × 10^5^ MC38 cells i.c. on day 3 p.i. (**E**–**H**) WT mice were injected with 5 × 10^5^ YMRG cells i.c., and lung tissue was analyzed for intracellular cytokine staining day 1.5 p.i. (E) Representative histogram and (F) quantification of IL-12p40/70 staining by percentage and mean fluorescence intensity (MFI) in DC1 and DC2. (G) Representative histogram and (H) quantification of IL-23p19 staining by percentage and MFI in DC1 and DC2. (**I**, **J**) Quantification of normalized metastatic burden in the lung of WT mice treated with IL-12p40 neutralizing Ab and injected with (I) 0.5 × 10^5^ YMR i.c. and (J) 1 × 10^5^ MC38 i.c. harvested on day 3 p.i. Data are representative of two or three independent experiments with at least (A–D, I, J) *n* = 5–8 mice and (E–H) *n* = 3 mice per group. Sample is compared using unpaired Student *t* test, and data are presented ± SEM. *p < 0.05, **p < 0.01, ***p < 0.001, ****p < 0.0001.

Because the immunological landscape surrounding pioneering metastatic cells can have both tumor-promoting and tumor-limiting characteristics (2, 3), we investigated the consequence of depleting the observed immune cells interacting with metastatic YMR in the lung. We generated CD11c^DTR^ and CD11b^DTR^ BMC mice that, following the i.t. administration of DT, resulted in the lung-targeted ablation of myeloid cells and activated NK cells (hereafter referred to as CD11c^Δ^ and CD11b^Δ^) (Supplemental Fig. 2A) (29). Targeting of CD11c- and CD11b-expressing immune populations resulted in an approximately fourfold increase in bioluminescence signal in the lung compared with WT controls on day 3 p.i. (Fig. 2A, Supplemental Fig. 2B), indicating a requirement for CD11c^+^ and CD11b^+^ immune cells in limiting initial metastatic burden.

Analysis of CCR2^−/−^ mice, which target the recruitment of monocytes, or DT-treated Mgl2^DTR^ and CD169^DTR^ mice, which deplete Mgl2-expressing interstitial macrophages and DC2 or CD169^+^ alveolar macrophages, respectively (hereafter referred to as Mgl2^Δ^ and CD169^Δ^) (Supplemental Fig. 2A, 2C), bearing YMR metastases did not result in increased burden in the lung on day 3 after i.c. injection (Fig. 2B; Supplemental Fig. 2B, 2D), thus suggesting that the recruitment of monocytes or Mgl2- and CD169-expressing cells is broadly dispensable for conferring initial control of lung metastasis. To determine whether DCs, the remaining major population of CD11c- and CD11b-expressing cells in the lung, can confer early metastatic protection, we injected immune-sufficient WT and DC1- and DC2-depleted mice (Zbtb46^DTR^ BMC) or DC1-depleted mice (XCR1^DTR^ mice) (referred to hereafter as DC^Δ^ and XCR1^Δ^, respectively) (Supplemental Fig. 2A) with YMR i.c. following i.t. DT administration. Early metastatic burden in the lung was significantly increased in DC^Δ^ mice compared with WT or XCR1^Δ^ mice (Fig. 2C, Supplemental Fig. 2E), suggesting that DC2, but not DC1, are required to mediate early metastatic control in the lung. Additionally, we did not observe an increase in metastatic burden in Mgl2^Δ^ mice (Fig 2B; Supplemental Fig. 2C, 2D), suggesting that the Mgl2^+^ DC2 subset was not necessary to limit initial metastatic burden. Consistent with this, stereoscopic microscopy demonstrated an increase in GFP^+^ microclusters in whole lungs bearing YMR metastasis on day 3 p.i. in DC^Δ^ compared with WT mice (Supplemental Fig. 2F, 2G). Analysis of metastatic burden in a different cancer model, i.c. injection of luciferase-expressing colon cancer cell line MC38, and different route of metastatic dissemination, i.v. injection of YMR, similarly exhibited an increase in the initial metastatic burden of DC^Δ^ mice compared with WT or XCR1^Δ^ mice (Fig 2D, Supplemental Fig. 2H).

Notably, stimulated lung DC2s were the sole source of proinflammatory cytokines IL-12p40 and IL-23p19 at day 1.5 p.i. (Fig. 2E–2H). This initial source of proinflammatory cytokines was restricted to the lung and not observed in DC subsets harvested from the spleen of metastasis-bearing mice (Supplemental Fig. 2I), suggesting that lung DC2, not DC1, were selectively activated early during initial metastatic seeding. IL-12p40 neutralizing Ab treatment resulted in increased initial metastatic burden (Fig. 2I, 2J; Supplemental Fig. 2J), suggesting that DC2 control of pioneering metastatic cells occurred in an IL-12p40-dependent manner. Because i.t. administration of DT resulted in the targeted ablation of lung DC but not splenic or lymph node DCs (Supplemental Fig. 2K), these results collectively suggest that local lung DC2 are required to limit metastatic burden following initial tissue seeding in an IL-12p40-dependent manner.

### Dendritic cell–intrinsic IRF3/7 signaling is required to limit initial metastatic burden

**FIGURE 3.**
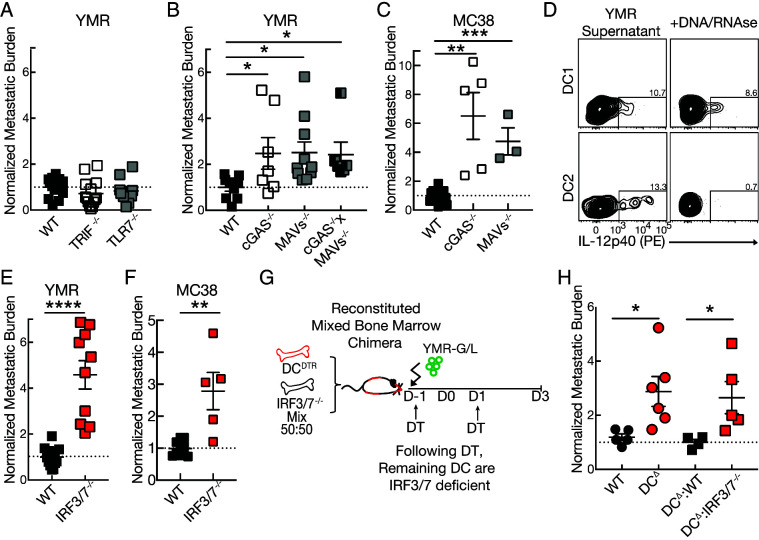
DC-intrinsic IRF3/7 signaling is required for early antimetastatic control. (**A**, **B**) Quantification of normalized metastatic burden in the lung of (A) indicated endosomal nucleic acid (NA) sensor–deficient mice, (B) indicated cytosolic NA sensor–deficient mice injected with 0.5 × 10^5^ YMR i.c. and harvested day 3 p.i. (**C**) Quantification of normalized metastatic burden in the lung of indicated cytosolic NA sensor–deficient mice injected with 1 × 10^5^ MC38 i.c. and harvested on day 3 p.i. (**D**) Quantification of IL-12p40 from sort-purified naive lung DC1 and DC2 incubated in vitro with YMR-derived cell media treated with DNase and RNase. (**E**, **F**) Quantification of normalized metastatic burden in the lung of transcription factor IRF3- and IRF7-knockout mice injected with (E) 0.5 × 10^5^ YMR and (F) 1 × 10^5^ MC38 i.c. and harvested on day 3 p.i. (**G**) Schematic of experiment. Lethally irradiated WT mice were reconstituted with either WT or IRF3/7^−/−^ bone marrow mixed 50:50 with DC^DTR^ bone marrow. Following reconstitution, mice were challenged with 0.5 × 10^5^ YMR i.c. One day before and after metastatic inoculation, mice were treated with DT i.t. (**H**) Quantification of normalized metastatic burden in the lung of indicated BMC mice on day 3 p.i. Data are representative of (A–C, E, F) three and (D, H) two independent experiments with at least *n* = 3–5 mice per group. Sample is compared using unpaired Student *t* test, and data are presented as mean ± SEM. *p < 0.05, **p < 0.01, ***p < 0.001, ****p < 0.0001.

Previous studies have demonstrated that sensing of tumor-derived nucleic acids by innate immune cells is critical to induce both endogenous and therapeutic antitumor immune responses (30–32). Because chromosomal instability and dysregulation of nucleic acids are hallmarks of metastatic disease progression (33, 34), we investigated whether sensing of nucleic acids is required to activate DC immune responses against pioneering metastatic cells in the lung. Although endosomal nucleic acid sensors were not required to limit initial metastatic burden evidenced by TLR7-deficient or TIR (Toll/IL-1R) domain-containing adapter inducing IFN-β–deficient (downstream of TLR3 signaling) mice (Fig. 3A), mice deficient in DNA and RNA cytosolic sensors were impaired in metastatic protection, as *Mb21d*^−/−^ (cGas^−/−^), *Mavs*^−/−^, and cGas*^−/−^Mavs*^−/−^ mice showed at least a doubling of lung metastatic burden on day 3 p.i. (Fig. 3B, 3C). To further interrogate the contribution of each DC subset, we stimulated FACS-sorted, purified, naive lung DC2 and DC1 with YMR media supernatant that had been treated in vitro with DNase and RNase. Although untreated YMR media stimulated both DC2 and DC1 to produce IL-12p40, media treatment with nucleases abrogated cytokine production only in DC2 (Fig. 3D), suggesting that DC2 are responsible for sensing tumor cells via nucleic acid sensors. Remarkably, mice deficient in transcription factors IRF3 and IRF7 downstream of canonical nucleic acid sensing signaling pathways exhibited significant increased lung metastatic burden on day 3 after i.c. injection (Fig. 3E, 3F). To determine if IRF3/7 signaling was DC intrinsic, we generated mixed BMC mice reconstituted with DC^DTR^ and *Irf3/7*^−/−^ bone marrow. Following DT treatment, the remaining DC are IRF3/7 deficient, thus generating DC-specific IRF3/7-deficient mice (Fig. 3G). Notably, DC^Δ^:*Irf3/7*^−/−^ BMC exhibited a similarly impaired ability to limit metastatic YMR burden in the lung compared with total DC^Δ^ mice (Fig. 3H). These findings suggest that DC-intrinsic activation of transcription factor IRF3/7 by cytosolic nucleic acid sensors is necessary to initiate DC-mediated control of pioneering metastatic cells.

### DC2 directs IFN-γ production by extravascular NK cells to confer early antimetastatic protection

**FIGURE 4.**
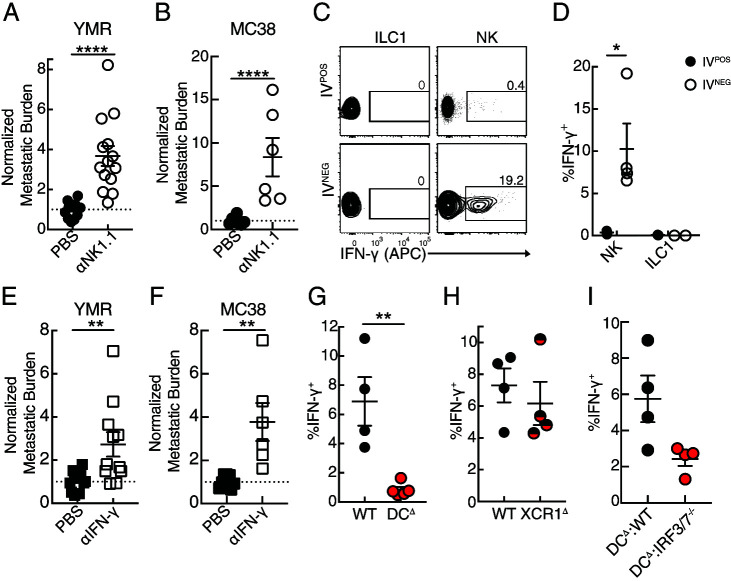
DC2 direct robust production of IFN-γ by extravascular NK cells to limit early metastatic burden. (**A**, **B**) Quantification of normalized metastatic burden in the lung of WT mice treated with α-NK1-depleting Ab and injected with (A) 0.5 × 10^5^ YMR and (B) 1 × 10^5^ MC38 i.c. and harvested on day 3 p.i. (**C**) Representative flow cytometric plots and (**D**) quantification of intracellular staining of IFN-γ CD45.2 i.v. (IV) labeled and unlabeled fraction in the indicated lymphocyte population in the lungs of WT mice injected with 5 × 10^5^ YMR-G/L i.c. harvested on day 3 p.i. (T cell = CD3^+^,TCRαβ^+^,NK1.1^−^,DX5^−^; ILC1 = CD3^−^,NK1.1^+^,DX5^−^,Eomes^−^,CD200R^+^; NK = CD3^−^,NK1.1^+^, DX5^+^,Eomes^+^,CD200R^−^; NKT = NK1.1^+^,CD3^+^,TCRαβ^+^). (**E**, **F**) Quantification of normalized metastatic burden in the lungs of WT mice treated with IFN-γ-blocking Ab and injected with (E) 0.5 × 10^5^ YMR and (F) 1 × 10^5^ MC38 i.c. and harvested on day 3 p.i. (**G**–**I**) Quantification of intracellular IFN-γ staining in IV^−^ NK cells from the lungs of DC^DTR^ BMC (G), XCR1^DTR^ (H), and DC^DTR^:IRF3/7^−/−^ BMC (I) mice injected with 5 × 10^5^ YMR-G/L i.c., treated with DT i.t., and harvested on day 3 p.i. Data are representative of at least three independent experiments with (A, B, E–I) *n* = 4–8 mice and (C, D) *n* = 3 mice per group. Sample is compared using unpaired Student *t* test, and data are presented as mean ± SEM. *p < 0.05, **p < 0.01, ****p < 0.0001.

It is well documented that DCs are critical mediators in translating various tissue insults to activate adaptive lymphocytes and ILCs during host antiviral and antitumor responses (9, 35). Thus, the requirement of DC2 in limiting early metastatic burden in the lung raised the possibility that DC2 could be potentiating effector lymphocyte responses that confer host antimetastatic immunity. Mice with germline deficiency in B cells, αβ T cells, and γδ T cells did not show increased metastatic burden in the lung compared with WT controls day 3 after i.c. injection (Supplemental Fig. 3A–3C). Consistent with these results, systemic Ab depletion of CD4^+^ T cells and CD8^+^ T cells showed metastatic burden comparable to that of PBS-treated mice (Supplemental Fig. 3A–3D). Although RAG2^−/−^ mice do succumb to eventual end-stage metastatic disease (Supplemental Fig. 3E, 3F), these results indicate that adaptive lymphocytes are not required to limit initial metastatic growth within the first 3 d of metastatic seeding. Notably, Ab-mediated ablation of group 1 ILCs resulted in a drastic increase in initial lung metastatic burden (Fig. 4A, 4B; Supplemental Fig. 3D, 3G), indicating that NK1.1^+^ group 1 ILC responses are a component of the initial host antimetastatic immunity program.

Because group 1 ILC effector functions are defined by their ability to rapidly produce IFN-γ (36, 37), we investigated if IFN-γ was required to limit initial metastatic burden. We found that among group 1 ILC subsets, only NK cells produced robust amounts of IFN-γ in vivo after early metastatic seeding in the lung (Fig. 4C, 4D). This initial IFN-γ response was restricted to the extravascular portion of lung lymphocytes in metastasis-bearing mice and was not observed in peripheral tissues (Fig. 4C, 4D; Supplemental Fig. 3H–3J). Additionally, IFN-γ-neutralizing treatment resulted in increased initial metastatic burden in a similar manner to NK1.1^+^ depleted mice (Fig. 4E, 4F; Supplemental Fig. 3K), suggesting that NK cell control of pioneering metastatic cells in the lung occurred in an IFN-γ-dependent manner local to the lung. Next, we evaluated the requirement for DC2 in potentiating NK cell IFN-γ responses. Production of IFN-γ by extravascular NK cells was abrogated in DC^Δ^ and in DC^Δ^:*Irf3/7*^−/−^ BMC mice but not in XCR1^Δ^ mice (Fig. 4G–4I), indicating that DC2, not DC1, was responsible for mediating local NK cell effector function to limit initial metastatic burden in an IRF3/7-dependent manner.

**FIGURE 5.**
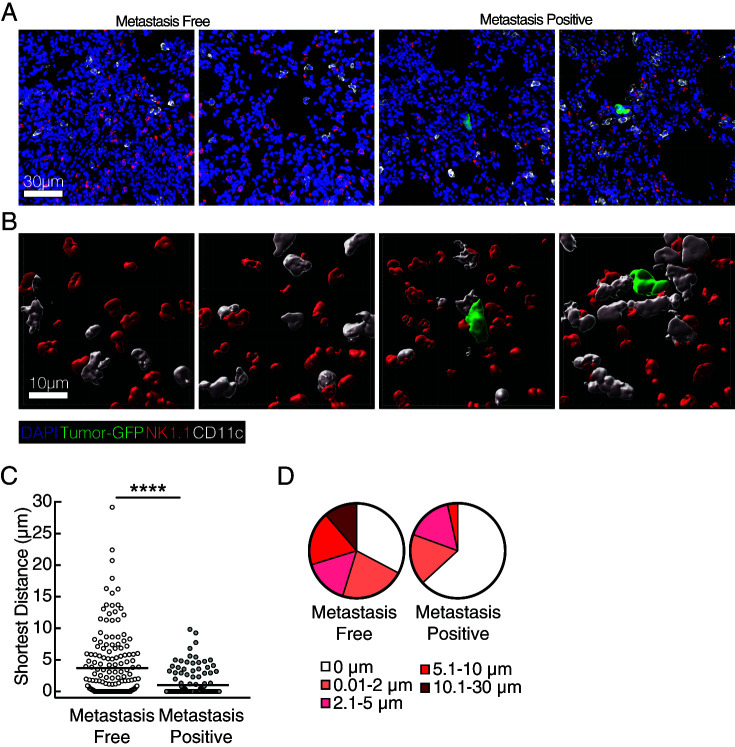
DC and NK cells colocalize around pioneering metastatic cells. WT mice were injected with 5 × 10^5^ YMR-G/L i.c. and analyzed by confocal microscopy on day 3 p.i. (**A**) Representative confocal image of frozen lung section depicting GFP^+^ YMR-G/L cell (green) NK1.1-stained cells (red), CD11c-stained cells (white), and costained with DAPI (blue). (**B**) Imaris rendering of three-dimensional surface interactions between GFP^+^ tumor cell, CD11c^+^ cell, and NK1.1^+^ cell from magnified section in (A). (**C**) Quantification of the shortest distance and (**D**) Frequency parsed by shortest distance between CD11c^+^ and NK1.1^+^ surfaces in metastasis-free and metastasis-positive sections. Data are representative of two independent experiments with *n* = 3 mice per group. Quantification data are from 120 metastasis-free sections and 30 metastasis-positive sections. Sample is compared using unpaired Student *t* test, and data are presented as mean ± SEM. ****p < 0.0001.

We next sought to characterize the nature of DC2 and NK cell localization relative to pioneering metastatic cells during the early stage of metastatic progression in the lung. Confocal microscopy identified clusters of CD11c^+^ and NK1.1^+^ that colocalized around pioneering metastatic cells in the lung (Fig. 5A, 5B). Notably, CD11c^+^ and NK1.1^+^ cells were located closer to each other in metastasis-positive sections than in metastasis-free sections, with an average distance of 1.0 μm compared with 3.9 μm (Fig. 5C). Additionally, we observed that the majority of NK1.1^+^ and CD11c^+^ were in direct physical contact (shortest distance of 0 μm apart) in metastasis-positive sections (Fig. 5D). This contrasts with metastasis-free sections, where the majority of NK1.1^+^ and CD11c^+^ cells were not in direct contact with each other and a significant proportion of cells were at least 10 μm apart from each other, a characteristic not observed in metastasis-positive sections (Fig. 5D). Collectively, these results suggest that DC2 and NK cells colocalize near pioneering, metastatic cells and orchestrate a nonredundant immune response to limit initial metastatic burden.

### DC2 signature is correlated with NK cell and IFN-γ responses in human metastasis patients

In order to evaluate the clinical relevance of our findings, we examined the association of human DC2 with NK cells and IFN-γ in patients with metastatic solid tumors. Using publicly available transcriptomic analysis (24, 25), we identified a unique DC2-specific signature comprising *IRF4*, *CD1A*, *CD1C*, and *CLEC10A* and an NK cell–specific signature comprising *NCR1*, *KLRD1*, *KLRF1*, *KLRC3*, and *KIR2DL4* to assess the presence of each immune population. By analyzing the MET500 bulk transcriptomic dataset (38) (see *Materials and Methods*), we found that the level of DC2 signature expression correlated with NK cell signature in the lung, independent of primary tumor origin (*R* = 0.62) (Supplemental Fig. 4A). Additionally, our identified DC2 exhibited a strong correlation with *IFNG* in metastasis from the lung (*R* = 0.51) (Supplemental Fig. 4B). As a control comparison, we observed a weak correlation between either the NK cell signature or *IFNG* expression against a monocyte/macrophage signature in the lung (*R* = 0.16 and 0.047, respectively) (Supplemental Fig. 4C, 4D), consistent with prior reports (3, 39). Collectively, our results suggest that a similar axis of DC2, NK cells, and IFN-γ exists in sites of metastatic dissemination.

## Discussion

Here, we demonstrated an essential role for the DC2–NK cell axis that limits initial metastatic burden via the robust production of IL-12p40 and IFN-γ in two different cancer models, melanoma and colon cancer, following different routes of metastatic dissemination. Our data demonstrated that the ablation of both DC1 and DC2 resulted in increased metastatic burden and abrogation of NK-derived IFN-γ, which was not phenocopied when only DC1 were ablated. This indicated that DC1 are dispensable for initial protection, whereas DC2 are required to mediate initial metastatic control. Furthermore, ex vivo cytokine analysis demonstrated that DC2, not DC1, are the main source of the key proinflammatory cytokine IL-12p40/70 and IL-23p19 in response to sensing tumor-derived nucleic acids, the latter of which is a potent activator of IFN-γ production in NK cells (23). Although it is possible that other cells in the metastatic niche could be involved in translating DC2-derived signals to activate antitumor NK cells, analysis of metastatic burden in different knockout and cell ablation models and the short time frame of our observations strongly suggest that this circuit is restricted to DC2 and NK cells. Because this preceded the recruitment of adaptive T cell responses, our results revealed an innate immune control of metastatic tumors that occurs within the first few days of seeding within the lung. Thus, prior to the onset of adaptive immunity, DC2-directed NK cell responses represent a nonredundant effector mechanism to directly sense and target pioneering, metastatic cells in the tissue and confer early antimetastatic immunity.

Numerous preclinical studies have reported the important role of NK cells in controlling metastatic diseases (5, 40). Previous studies have implicated circulating NK cells in targeting circulating latent cancer cells or preventing the escape of metastatic cancer cells from primary tumor cells (6). Our study reveals that NK cells adopt an additional role in antimetastatic immunity whereby extravascular NK cells present in the lung play a critical role in the control of pioneering metastatic cells upon seeding within the local tissues. Notably, NK cells, not their tissue-resident counterpart ILC1, serve as the robust source of IFN-γ that limits the initial metastatic burden. Ablation of DCs in DC^Δ^ mice did not affect NK cell number during the first 3 d of metastatic growth in the lung, but it did affect the ability of NK cells to produce IFN-γ. This suggests that DCs do not play a role in the recruiting of NK cells but rather orchestrate their local antitumor effector responses. However, this does not rule out the possibility that DCs play a role in the localized recruitment or maintenance of extravascular NK cells around individual tumor cells. Although our data indicate that DC1 are dispensable for initial control of metastatic burden, DC1 are required to present Ag to T cells and provide local support to T cells in the tumor microenvironment (8, 41). Thus, we speculate that NK cells could play a role in mediating a later-stage antimetastatic T cell immune response, either by recruiting DC1 by secreting CXCL1 (as is documented in other tumor models [42]) or by triggering the production of IFN-γ-inducible chemokines such as CXCL9 and CXCL10, critical for recruitment of effector T cells. How NK cells serve an additional role in directing DC1-mediated late-stage adaptive T cell responses in the metastatic niche requires further investigation.

Our results extend the role of tissue-resident DCs as local sentinels orchestrating immune responses previously described in settings of pathogenic challenge to initial antimetastatic responses. The heterogeneity of DC responses is usually defined by its ability to direct specific types of effector programs (9, 21). Specifically, DC1 primarily direct type 1 immune responses, such as the rapid activation of ILC1 cells at barrier sites and preferential ability to prime CD8^+^ T cell and CD4^+^ Th1 responses during host antitumor and antiviral responses (9, 43). Additionally, checkpoint blockade and cytokine-based cancer immunotherapies require DC1 in mediating effective CD8^+^ T cell antitumor immunity (41, 44, 45). Conversely, subsets of DC2 predominantly govern types 2 and 3 immune responses against parasites by activating ILC2 and Th2 or against extracellular bacteria by activating ILC3 and Th17 (20, 22, 46, 47). However, our data suggest that a more fluid paradigm exists whereby DC2 can also be critical in orchestrating a type 1 NK cell–mediated IFN-γ production during host antimetastatic responses. This is consistent with recent reports highlighting the ability of DC2 to robustly produce type 1 stimulating cytokine IL-12 and sufficiently prime CD8^+^ T cell responses during a respiratory viral challenge in the lung, as well as polarize antitumor Th1 cells in a s.c. model of murine melanoma (15, 48, 49). Given the heterogeneity of DC2 during homeostasis and disease states, whether the ability of DC2 to adapt effector responses to specific cues is due to cellular plasticity or to discrete DC2 subsets remains to be fully elucidated.

Analysis of human datasets revealed a correlation of DC2 and NK cells among different metastases independent of the primary tumor source. Thus, it is possible that a similar circuit exists in humans. Chromatin and single-cell transcriptomic analysis have demonstrated that DC2 are found and conserved in both humans and mice, with a shared expression of surface markers, production of effector molecules, and host immune functions (24, 50). Notably, recent reports have demonstrated that tumor DC2 can acquire regulatory features that limit antitumor immunity that is found in both humans and mice (49). Although our model suggests an early protective role for DC2, the extent to which this is shared in humans and how DC2 transition from a protective to a suppressive role remain to be elucidated.

Currently, the genetic tools to target lung DC2 are limited. A recent study highlighted the targeting of *Zeb2* enhancers to deplete peripheral DC2. However, this model additionally targets monocytes (51). Consistent with previous studies, we observed that DC-specific deletion of *Irf4* in mice (*Zbtb46*^Cre/+^x*Irf4^fl/fl^*) did not result in decreased lung DC2 numbers but did impact DC1 and DC2 numbers in the mesenteric and mediastinal lymph node (52; O.-E. Weizman and A. Iwasaki, unpublished observations). This likely reflects the nature of IRF4 expression after the development of committed DC2 and may play an active role in mediating DC migration (53, 54). Additionally, IRF4 has recently been identified in a subset of IRF8-expressing DC1 during antitumor response, undermining its utility as a DC2-defining transcription factor (49).

Although our observations are specific to metastatic seeding of the lung, due to its vascularized nature, the lung represents a prime tissue environment for metastatic seeding for several primary tumors and is a source for further metastatic dissemination to other locations (55–57). However, it is likely that due to the unique immune landscape that defines distal tissues and the different immunogenic capacities of disseminated cancer cells, different tissue-specific DC–effector cell immune circuits exist to serve a similar nonredundant function of limiting initial metastatic burden before the recruitment of circulating antimetastatic responses. Additionally, similar DC–effector circuits could be deployed to mediate control of initial aberrant transformation of primary tumors and thus warrant future investigation.

Although it is estimated that 90% of all the morbidity and mortality of cancer is due to metastases (58), only a limited number of existing therapeutic anticancer portfolios target metastasis. Current efforts to leverage DC to mediate antitumor efficacy focus on autologous DC and vaccination approaches to prime the effector T cell response (59) and have met with limited success. Taken together, our work highlights, to our knowledge, a novel DC2–NK cell axis that suggests improved targeting of DC-mediated NK cell activation may provide an untapped avenue for novel therapeutic targets.

## Supplementary Material

Supplemental 1 (PDF)

## References

[1] Valastyan, S., R. A. Weinberg. 2011. Tumor metastasis: molecular insights and evolving paradigms. Cell 147: 275–292. 22000009 10.1016/j.cell.2011.09.024 PMC3261217

[2] Massagué, J., A. C. Obenauf. 2016. Metastatic colonization by circulating tumour cells. Nature 529: 298–306. 26791720 10.1038/nature17038 PMC5029466

[3] Kitamura, T., B. Z. Qian, J. W. Pollard. 2015. Immune cell promotion of metastasis. Nat. Rev. Immunol. 15: 73–86. 25614318 10.1038/nri3789 PMC4470277

[4] Qian, B. Z., J. Li, H. Zhang, T. Kitamura, J. Zhang, L. R. Campion, E. A. Kaiser, L. A. Snyder, J. W. Pollard. 2011. CCL2 recruits inflammatory monocytes to facilitate breast-tumour metastasis. Nature 475: 222–225. 21654748 10.1038/nature10138 PMC3208506

[5] López-Soto, A., S. Gonzalez, M. J. Smyth, L. Galluzzi. 2017. Control of metastasis by NK cells. Cancer Cell 32: 135–154. 28810142 10.1016/j.ccell.2017.06.009

[6] Malladi, S., D. G. Macalinao, X. Jin, L. He, H. Basnet, Y. Zou, E. de Stanchina, J. Massagué. 2016. Metastatic latency and immune evasion through autocrine inhibition of WNT. Cell 165: 45–60. 27015306 10.1016/j.cell.2016.02.025 PMC4808520

[7] Lorenzo-Herrero, S., A. López-Soto, C. Sordo-Bahamonde, A. P. Gonzalez-Rodriguez, M. Vitale, S. Gonzalez. 2018. NK cell-based immunotherapy in cancer metastasis. Cancers (Basel) 11: 29. 30597841 10.3390/cancers11010029 PMC6357056

[8] Headley, M. B., A. Bins, A. Nip, E. W. Roberts, M. R. Looney, A. Gerard, M. F. Krummel. 2016. Visualization of immediate immune responses to pioneer metastatic cells in the lung. Nature 531: 513–517. 26982733 10.1038/nature16985 PMC4892380

[9] Durai, V., K. M. Murphy. 2016. Functions of murine dendritic cells. Immunity 45: 719–736. 27760337 10.1016/j.immuni.2016.10.010 PMC5145312

[10] Merad, M., P. Sathe, J. Helft, J. Miller, A. Mortha. 2013. The dendritic cell lineage: ontogeny and function of dendritic cells and their subsets in the steady state and the inflamed setting. Annu. Rev. Immunol. 31: 563–604. 23516985 10.1146/annurev-immunol-020711-074950 PMC3853342

[11] Dress, R. J., A. Y. Wong, F. Ginhoux. 2018. Homeostatic control of dendritic cell numbers and differentiation. Immunol. Cell Biol. 96: 463–476. 29473216 10.1111/imcb.12028

[12] Bosteels, C., C. L. Scott. 2020. Transcriptional regulation of DC fate specification. Mol. Immunol. 121: 38–46. 32151907 10.1016/j.molimm.2020.02.021 PMC7187805

[13] Guilliams, M., C. A. Dutertre, C. L. Scott, N. McGovern, D. Sichien, S. Chakarov, S. Van Gassen, J. Chen, M. Poidinger, S. De Prijck, . 2016. Unsupervised high-dimensional analysis aligns dendritic cells across tissues and species. Immunity 45: 669–684. 27637149 10.1016/j.immuni.2016.08.015 PMC5040826

[14] Gutiérrez-Martínez, E., R. Planès, G. Anselmi, M. Reynolds, S. Menezes, A. C. Adiko, L. Saveanu, P. Guermonprez. 2015. Cross-presentation of cell-associated antigens by MHC class I in dendritic cell subsets. Front. Immunol. 6: 363. 26236315 10.3389/fimmu.2015.00363 PMC4505393

[15] Binnewies, M., A. M. Mujal, J. L. Pollack, A. J. Combes, E. A. Hardison, K. C. Barry, J. Tsui, M. K. Ruhland, K. Kersten, M. A. Abushawish, . 2019. Unleashing type-2 dendritic cells to drive protective antitumor CD4^+^ T cell immunity. Cell 177: 556–571.e16. 30955881 10.1016/j.cell.2019.02.005 PMC6954108

[16] Bedoui, S., P. G. Whitney, J. Waithman, L. Eidsmo, L. Wakim, I. Caminschi, R. S. Allan, M. Wojtasiak, K. Shortman, F. R. Carbone, . 2009. Cross-presentation of viral and self antigens by skin-derived CD103^+^ dendritic cells. Nat. Immunol. 10: 488–495. 19349986 10.1038/ni.1724

[17] Broz, M. L., M. Binnewies, B. Boldajipour, A. E. Nelson, J. L. Pollack, D. J. Erle, A. Barczak, M. D. Rosenblum, A. Daud, D. L. Barber, . 2014. Dissecting the tumor myeloid compartment reveals rare activating antigen-presenting cells critical for T cell immunity. [Published erratum appears in 2014 *Cancer Cell* 26: 938.] Cancer Cell 26: 638–652. 25446897 10.1016/j.ccell.2014.09.007 PMC4254577

[18] Gao, Y., S. A. Nish, R. Jiang, L. Hou, P. Licona-Limón, J. S. Weinstein, H. Zhao, R. Medzhitov. 2013. Control of T helper 2 responses by transcription factor IRF4-dependent dendritic cells. Immunity 39: 722–732. 24076050 10.1016/j.immuni.2013.08.028 PMC4110745

[19] Krishnaswamy, J. K., U. Gowthaman, B. Zhang, J. Mattsson, L. Szeponik, D. Liu, R. Wu, T. White, S. Calabro, L. Xu, . 2017. Migratory CD11b^+^ conventional dendritic cells induce T follicular helper cell-dependent antibody responses. Sci. Immunol. 2: eaam9169. 29196450 10.1126/sciimmunol.aam9169 PMC7847246

[20] Kumamoto, Y., M. Linehan, J. S. Weinstein, B. J. Laidlaw, J. E. Craft, A. Iwasaki. 2013. CD301b^+^ dermal dendritic cells drive T helper 2 cell-mediated immunity. Immunity 39: 733–743. 24076051 10.1016/j.immuni.2013.08.029 PMC3819035

[21] Briseño, C. G., T. L. Murphy, K. M. Murphy. 2014. Complementary diversification of dendritic cells and innate lymphoid cells. Curr. Opin. Immunol. 29: 69–78. 24874447 10.1016/j.coi.2014.04.006 PMC5161034

[22] Satpathy, A. T., C. G. Briseño, J. S. Lee, D. Ng, N. A. Manieri, W. Kc, X. Wu, S. R. Thomas, W. L. Lee, M. Turkoz, . 2013. Notch2-dependent classical dendritic cells orchestrate intestinal immunity to attaching-and-effacing bacterial pathogens. Nat. Immunol. 14: 937–948. 23913046 10.1038/ni.2679 PMC3788683

[23] Weizman, O. E., N. M. Adams, I. S. Schuster, C. Krishna, Y. Pritykin, C. Lau, M. A. Degli-Esposti, C. S. Leslie, J. C. Sun, T. E. O’Sullivan. 2017. ILC1 confer early host protection at initial sites of viral infection. Cell 171: 795–808.e12. 29056343 10.1016/j.cell.2017.09.052 PMC5687850

[24] Brown, C. C., H. Gudjonson, Y. Pritykin, D. Deep, V. P. Lavallée, A. Mendoza, R. Fromme, L. Mazutis, C. Ariyan, C. Leslie, . 2019. Transcriptional basis of mouse and human dendritic cell heterogeneity. Cell 179: 846–863.e24. 31668803 10.1016/j.cell.2019.09.035 PMC6838684

[25] Cursons, J., F. Souza-Fonseca-Guimaraes, M. Foroutan, A. Anderson, F. Hollande, S. Hediyeh-Zadeh, A. Behren, N. D. Huntington, M. J. Davis. 2019. A gene signature predicting natural killer cell infiltration and improved survival in melanoma patients. Cancer Immunol. Res. 7: 1162–1174. 31088844 10.1158/2326-6066.CIR-18-0500

[26] Wilk, A. J., A. Rustagi, N. Q. Zhao, J. Roque, G. J. Martínez-Colón, J. L. McKechnie, G. T. Ivison, T. Ranganath, R. Vergara, T. Hollis, . 2020. A single-cell atlas of the peripheral immune response in patients with severe COVID-19. Nat. Med. 26: 1070–1076. 32514174 10.1038/s41591-020-0944-y PMC7382903

[27] Khanna, C., K. Hunter. 2005. Modeling metastasis in vivo. Carcinogenesis 26: 513–523. 15358632 10.1093/carcin/bgh261

[28] Wang, J., C. J. Perry, K. Meeth, D. Thakral, W. Damsky, G. Micevic, S. Kaech, K. Blenman, M. Bosenberg. 2017. UV-induced somatic mutations elicit a functional T cell response in the YUMMER1.7 mouse melanoma model. Pigment Cell Melanoma Res. 30: 428–435. 28379630 10.1111/pcmr.12591 PMC5820096

[29] van Rijt, L. S., S. Jung, A. Kleinjan, N. Vos, M. Willart, C. Duez, H. C. Hoogsteden, B. N. Lambrecht. 2005. In vivo depletion of lung CD11c^+^ dendritic cells during allergen challenge abrogates the characteristic features of asthma. J. Exp. Med. 201: 981–991. 15781587 10.1084/jem.20042311 PMC2213109

[30] Flood, B. A., E. F. Higgs, S. Li, J. J. Luke, T. F. Gajewski. 2019. STING pathway agonism as a cancer therapeutic. Immunol. Rev. 290: 24–38. 31355488 10.1111/imr.12765 PMC6814203

[31] Jiang, X., V. Muthusamy, O. Fedorova, Y. Kong, D. J. Kim, M. Bosenberg, A. M. Pyle, A. Iwasaki. 2019. Intratumoral delivery of RIG-I agonist SLR14 induces robust antitumor responses. J. Exp. Med. 216: 2854–2868. 31601678 10.1084/jem.20190801 PMC6888973

[32] Demaria, O., S. Cornen, M. Daëron, Y. Morel, R. Medzhitov, E. Vivier. 2019. Harnessing innate immunity in cancer therapy. [Published erratum appears in 2019 *Nature* 576: E3.] Nature 574: 45–56. 31578484 10.1038/s41586-019-1593-5

[33] Bakhoum, S. F., B. Ngo, A. M. Laughney, J. A. Cavallo, C. J. Murphy, P. Ly, P. Shah, R. K. Sriram, T. B. K. Watkins, N. K. Taunk, . 2018. Chromosomal instability drives metastasis through a cytosolic DNA response. Nature 553: 467–472. 29342134 10.1038/nature25432 PMC5785464

[34] Chen, Q., A. Boire, X. Jin, M. Valiente, E. E. Er, A. Lopez-Soto, L. Jacob, R. Patwa, H. Shah, K. Xu, . 2016. Carcinoma–astrocyte gap junctions promote brain metastasis by cGAMP transfer. [Published erratum appears in 2017 *Nature* 544: 124.] Nature 533: 493–498. 27225120 10.1038/nature18268 PMC5021195

[35] Iwasaki, A., R. Medzhitov. 2010. Regulation of adaptive immunity by the innate immune system. Science 327: 291–295. 20075244 10.1126/science.1183021 PMC3645875

[36] Cortez, V. S., M. Colonna. 2016. Diversity and function of group 1 innate lymphoid cells. Immunol. Lett. 179: 19–24. 27394699 10.1016/j.imlet.2016.07.005 PMC5658203

[37] Riggan, L., A. G. Freud, T. E. O’Sullivan. 2019. True detective: unraveling group 1 innate lymphocyte heterogeneity. Trends Immunol. 40: 909–921. 31500958 10.1016/j.it.2019.08.005 PMC6823149

[38] Robinson, D. R., Y. M. Wu, R. J. Lonigro, P. Vats, E. Cobain, J. Everett, X. Cao, E. Rabban, C. Kumar-Sinha, V. Raymond, . 2017. Integrative clinical genomics of metastatic cancer. Nature 548: 297–303. 28783718 10.1038/nature23306 PMC5995337

[39] Richards, D. M., J. Hettinger, M. Feuerer. 2013. Monocytes and macrophages in cancer: development and functions. Cancer Microenviron. 6: 179–191. 23179263 10.1007/s12307-012-0123-x PMC3717063

[40] Chiossone, L., P. Y. Dumas, M. Vienne, E. Vivier. 2018. Natural killer cells and other innate lymphoid cells in cancer. [Published erratum appears in 2018 *Nat. Rev. Immunol.* 18: 726.] Nat. Rev. Immunol. 18: 671–688. 30209347 10.1038/s41577-018-0061-z

[41] Salmon, H., J. Idoyaga, A. Rahman, M. Leboeuf, R. Remark, S. Jordan, M. Casanova-Acebes, M. Khudoynazarova, J. Agudo, N. Tung, . 2016. Expansion and activation of CD103^+^ dendritic cell progenitors at the tumor site enhances tumor responses to therapeutic PD-L1 and BRAF inhibition. Immunity 44: 924–938. 27096321 10.1016/j.immuni.2016.03.012 PMC4980762

[42] Böttcher, J. P., E. Bonavita, P. Chakravarty, H. Blees, M. Cabeza-Cabrerizo, S. Sammicheli, N. C. Rogers, E. Sahai, S. Zelenay, C. Reis e Sousa. 2018. NK cells stimulate recruitment of cDC1 into the tumor microenvironment promoting cancer immune control. Cell 172: 1022–1037.e14. 29429633 10.1016/j.cell.2018.01.004 PMC5847168

[43] Mashayekhi, M., M. M. Sandau, I. R. Dunay, E. M. Frickel, A. Khan, R. S. Goldszmid, A. Sher, H. L. Ploegh, T. L. Murphy, L. D. Sibley, K. M. Murphy. 2011. CD8α^+^ dendritic cells are the critical source of interleukin-12 that controls acute infection by *Toxoplasma gondii* tachyzoites. Immunity 35: 249–259. 21867928 10.1016/j.immuni.2011.08.008 PMC3171793

[44] Zhou, T., W. Damsky, O. E. Weizman, M. K. McGeary, K. P. Hartmann, C. E. Rosen, S. Fischer, R. Jackson, R. A. Flavell, J. Wang, . 2020. IL-18BP is a secreted immune checkpoint and barrier to IL-18 immunotherapy. Nature 583: 609–614. 32581358 10.1038/s41586-020-2422-6 PMC7381364

[45] Böttcher, J. P., C. Reis e Sousa. 2018. The role of type 1 conventional dendritic cells in cancer immunity. Trends Cancer 4: 784–792. 30352680 10.1016/j.trecan.2018.09.001 PMC6207145

[46] Tussiwand, R., B. Everts, G. E. Grajales-Reyes, N. M. Kretzer, A. Iwata, J. Bagaitkar, X. Wu, R. Wong, D. A. Anderson, T. L. Murphy, . 2015. Klf4 expression in conventional dendritic cells is required for T helper 2 cell responses. Immunity 42: 916–928. 25992862 10.1016/j.immuni.2015.04.017 PMC4447135

[47] Lewis, K. L., M. L. Caton, M. Bogunovic, M. Greter, L. T. Grajkowska, D. Ng, A. Klinakis, I. F. Charo, S. Jung, J. L. Gommerman, . 2011. Notch2 receptor signaling controls functional differentiation of dendritic cells in the spleen and intestine. Immunity 35: 780–791. 22018469 10.1016/j.immuni.2011.08.013 PMC3225703

[48] Bosteels, C., K. Neyt, M. Vanheerswynghels, M. J. van Helden, D. Sichien, N. Debeuf, S. De Prijck, V. Bosteels, N. Vandamme, L. Martens, . 2020. Inflammatory type 2 cDCs acquire features of cDC1s and macrophages to orchestrate immunity to respiratory virus infection. Immunity 52: 1039–1056.e9. 32392463 10.1016/j.immuni.2020.04.005 PMC7207120

[49] Maier, B., A. M. Leader, S. T. Chen, N. Tung, C. Chang, J. LeBerichel, A. Chudnovskiy, S. Maskey, L. Walker, J. P. Finnigan, . 2020. A conserved dendritic-cell regulatory program limits antitumour immunity. [Published erratum appears in 2020 *Nature* 582: E17.] Nature 580: 257–262. 32269339 10.1038/s41586-020-2134-y PMC7787191

[50] See, P., C. A. Dutertre, J. Chen, P. Günther, N. McGovern, S. E. Irac, M. Gunawan, M. Beyer, K. Händler, K. Duan, . 2017. Mapping the human DC lineage through the integration of high-dimensional techniques. Science 356: eaag3009. 28473638 10.1126/science.aag3009 PMC7611082

[51] Liu, T. T., S. Kim, P. Desai, D. H. Kim, X. Huang, S. T. Ferris, R. Wu, F. Ou, T. Egawa, S. J. Van Dyken, . 2022. Ablation of cDC2 development by triple mutations within the Zeb2 enhancer. Nature 607: 142–148. 35732734 10.1038/s41586-022-04866-z PMC10358283

[52] Loschko, J., G. J. Rieke, H. A. Schreiber, M. M. Meredith, K. H. Yao, P. Guermonprez, M. C. Nussenzweig. 2016. Inducible targeting of cDCs and their subsets in vivo. J. Immunol. Methods 434: 32–38. 27073171 10.1016/j.jim.2016.04.004 PMC4902770

[53] Persson, E. K., H. Uronen-Hansson, M. Semmrich, A. Rivollier, K. Hägerbrand, J. Marsal, S. Gudjonsson, U. Håkansson, B. Reizis, K. Kotarsky, W. W. Agace. 2013. IRF4 transcription-factor-dependent CD103^+^CD11b^+^ dendritic cells drive mucosal T helper 17 cell differentiation. Immunity 38: 958–969. 23664832 10.1016/j.immuni.2013.03.009

[54] Schlitzer, A., N. McGovern, P. Teo, T. Zelante, K. Atarashi, D. Low, A. W. Ho, P. See, A. Shin, P. S. Wasan, . 2013. IRF4 transcription factor-dependent CD11b^+^ dendritic cells in human and mouse control mucosal IL-17 cytokine responses. Immunity 38: 970–983. 23706669 10.1016/j.immuni.2013.04.011 PMC3666057

[55] Nguyen, D. X., P. D. Bos, J. Massagué. 2009. Metastasis: from dissemination to organ-specific colonization. Nat. Rev. Cancer 9: 274–284. 19308067 10.1038/nrc2622

[56] Angelova, M., B. Mlecnik, A. Vasaturo, G. Bindea, T. Fredriksen, L. Lafontaine, B. Buttard, E. Morgand, D. Bruni, A. Jouret-Mourin, . 2018. Evolution of metastases in space and time under immune selection. Cell 175: 751–765.e16. 30318143 10.1016/j.cell.2018.09.018

[57] Steeg, P. S. 2016. Targeting metastasis. Nat. Rev. Cancer 16: 201–218. 27009393 10.1038/nrc.2016.25 PMC7055530

[58] Chaffer, C. L., R. A. Weinberg. 2011. A perspective on cancer cell metastasis. Science 331: 1559–1564. 21436443 10.1126/science.1203543

[59] Jahchan, N. S., A. M. Mujal, J. L. Pollack, M. Binnewies, V. Sriram, L. Reyno, M. F. Krummel. 2019. Tuning the tumor myeloid microenvironment to fight cancer. Front. Immunol. 10: 1611. 31402908 10.3389/fimmu.2019.01611 PMC6673698

