# Supplementary material for: Type 2 Dendritic Cells Orchestrate a Local Immune Circuit to Confer Antimetastatic Immunity
Source: J Immunol. 2023 Mar 3;210(8):1146–55. doi: 10.4049/jimmunol.2200697 (PMC10067787; doi:10.4049/jimmunol.2200697)
Supplement: Supplemental 1 (PDF) [file JI_2200697_Supplemental_1.pdf]

Supplemental Figure 1

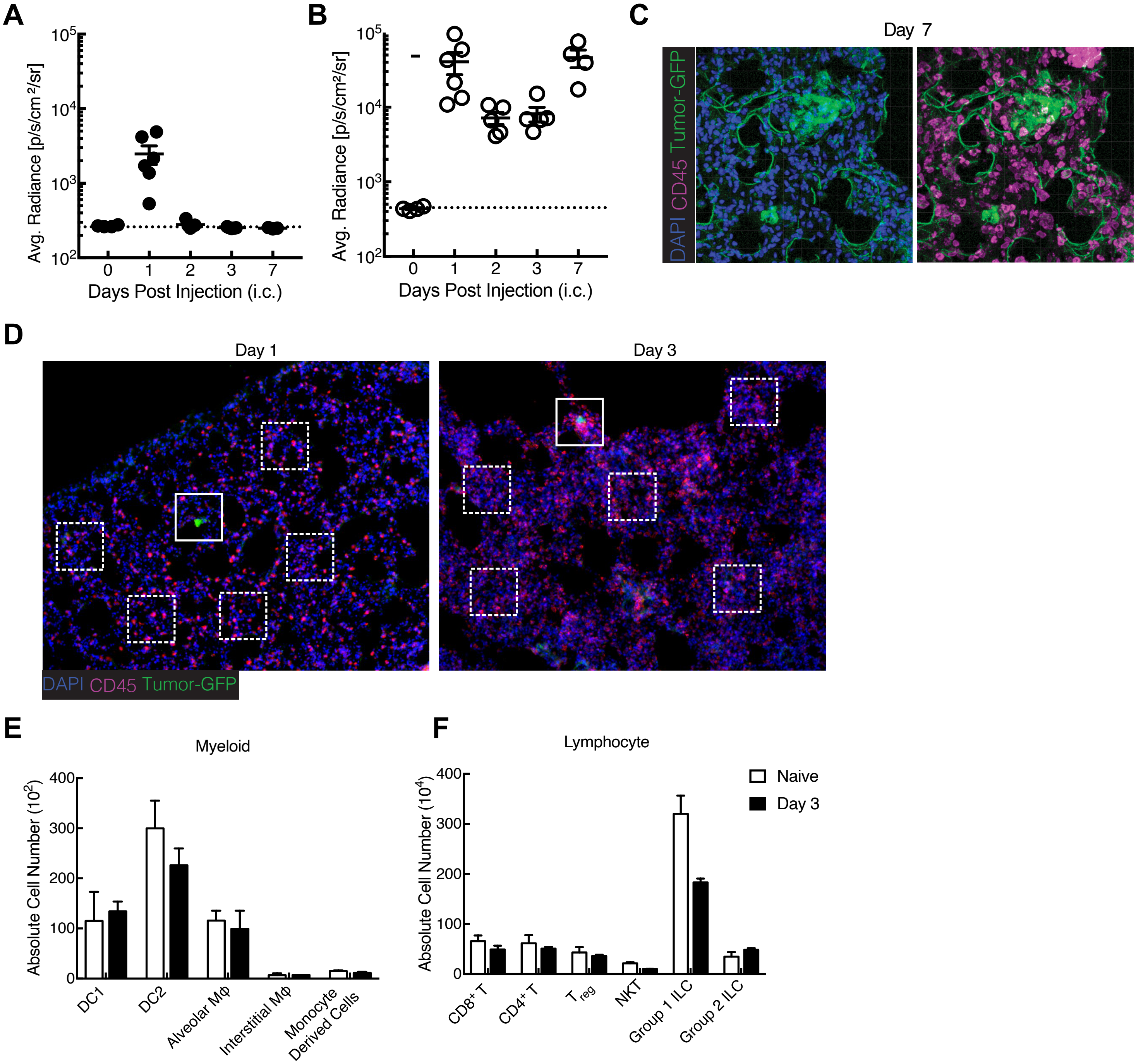

Supplemental Figure 1. Related to Figure 1. **Intracardiac model of lung metastasis results in the local recruitment of immune cells.**

Wild type (WT) mice were injected with $5x10^5$ YUMMER1.7 cells co-expressing GFP and luciferase (YMR) intracardially (i.c.). (A,B) Quantification of bioluminescence (average radiance) of whole spleen (A) and lung (B) at indicated days post injection (p.i.). (C) Representative confocal image of frozen lung section depicting YMR cell (GFP, green; left panel) colocalizing with CD45 stained cells (magenta; right panel) co-stained with DAPI (blue) at day 7 p.i. (D) Representative fluorescent microscopy image of frozen lung section depicting YMR GFP+, CD45, and DAPI stained cells at day 1 and 3 p.i.; used to make calculation in Fig. 1D. White solid line box indicates metastasis positive section, white dashed line box indicates metastasis free section. (E,F) Quantification of absolute cell number assed by flow cytometry of indicated extravascular (CD45.2 IV- fraction) myeloid (E) and lymphocyte (F) population in the lung day 3 post i.c. injection. (Myeloid Populations: DC1 = Lin-, CD64-, MHCIIhi, CD11chi, CD11b–, CD172a-, XCR1+; DC2 = Lin-, CD64-, MHCIIhi, CD11chi, CD11b+, CD172a+, XCR1-; Alveolar Macrophages (Φ) = Lin-, CD64+, CD169+; Interstitial MΦ = Lin-, CD64+, CD169-, CD11cneg, MHCIIhi, CD11b+; Monocyte Derived Cells = Lin-, CD64+, CD169-, CD11cint, MHCIIhi, CD11b+; Lin = TCRαβ+, CD3e+, NK1.1+, CD19+) . (Lymphocyte Populations: CD8+ T cells = CD19-, TCR αβ+, CD3e+, NK1.1-, CD8α+; CD4+ T cells = CD19-, TCRαβ+, CD3e+, NK1.1-, CD4+, Foxp3-; Treg cells = CD19-, TCRαβ+, CD3e+, NK1.1-, CD4+, Foxp3+;NKT = CD19-, TCRαβ+, CD3e+, NK1.1+; Group 1 ILC = CD19-, TCRαβ-, CD3e-, NK1.1+; Group 2 ILC = CD19-, TCRαβ-, CD3e-, NK1.1-, CD90+, KLRG1+) Data is representative of two independent experiments with (A,B) n=6 and (B-F) n=3 mice per group.

Supplemental Figure 2

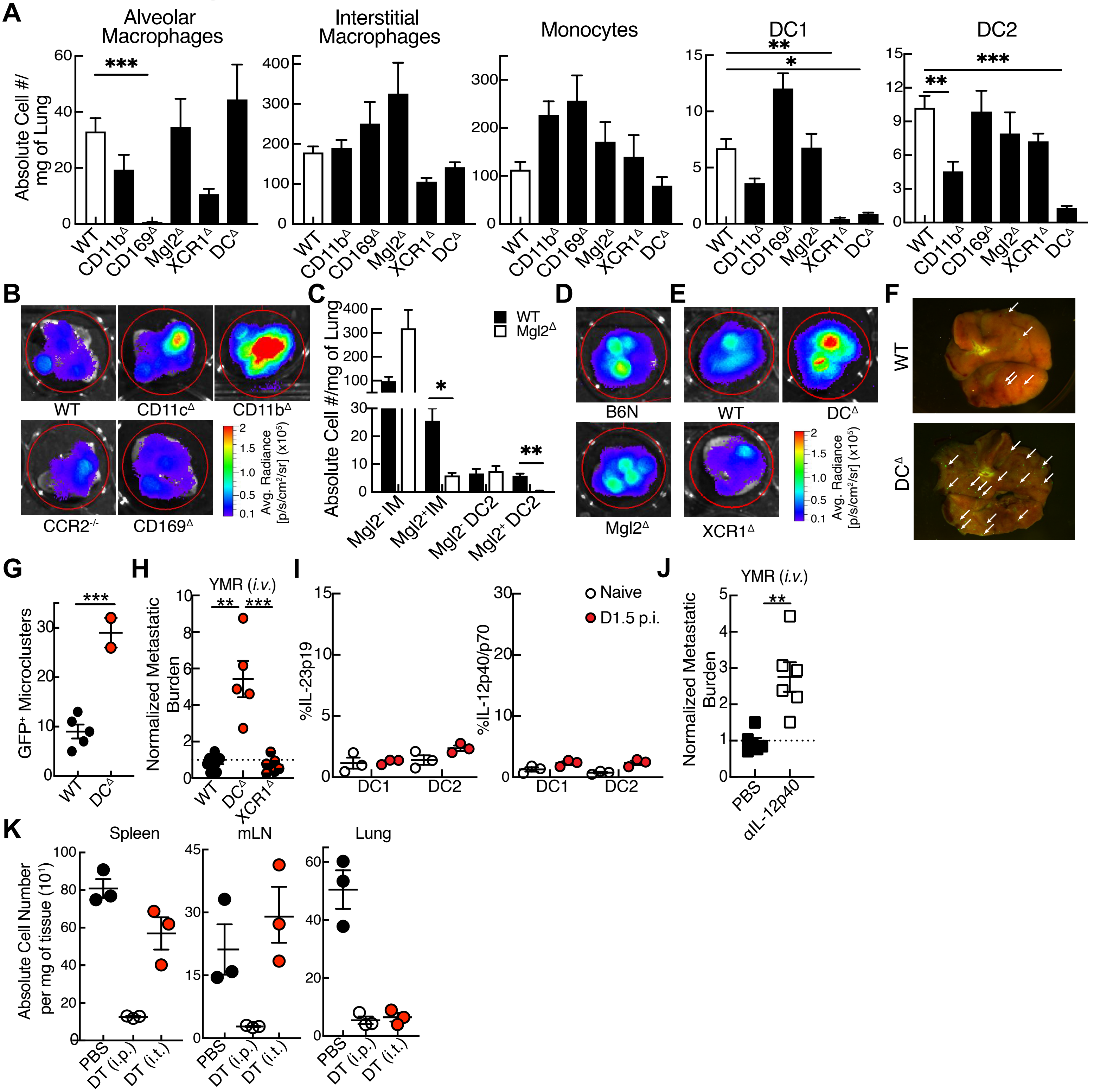

Supplemental Figure 2. Related to Figure 2. **Local DC2 limit initial metastatic burden in the lung.**
(A-G) Mice were injected with $0.5x10^5$ YMR cells i.c. and lung tissue was analyzed on D3 p.i. All mice were treated with diphtheria toxin (DT) intratracheally (i.t.) D-1 and D+1 post injection. (A) Quantification of absolute cell number assed by flow cytometry of indicated myeloid populations in indicated DTR mouse model. (B) Representative image of bioluminescence in indicated mouse model. (C) Quantification of absolute number of Mgl2 expressing cells in $Mgl2^{DTR}$ and WT (B6N) mice. (D,E) Representative image of bioluminescence in the lung in indicated mouse model. (F) Representative stereoscope image and (G) quantification of GFP+ micro-clusters (white arrows) from lung harvested from indicated mouse model. (H) Quantification of metastatic burden in the lung of indicated mouse model injected with $1x10^5$ YMR cells i.v. D3 p.i. (I) Quantification of intracellular IL-12p40/70 and IL-23p19 staining in splenic DC1 and DC2 from WT mice injected with $5x10^5$ YMR cells i.c. and analyzed D1.5 p.i. (J) Quantification of normalized metastatic burden in the lung of WT mice treated with IL-12p40 neutralizing antibody and injected with $1x10^5$ YMR i.v. harvested D3 p.i. (K) Quantification of absolute cell number total DC in spleen, lung, mesenteric lymph node (mLN) of D3 metastatic bearing DC^{DTR} mice treated with either PBS, DT intraperitoneally, or i.t. Data is representative of three independent experiments with at least (A-E,H,J) n=5 and (F,G,I,K) n=2-3 mice per group. Data is presented as ± SEM. (*p<0.05, **p<0.01,***p<0.001).

Supplemental Figure 3

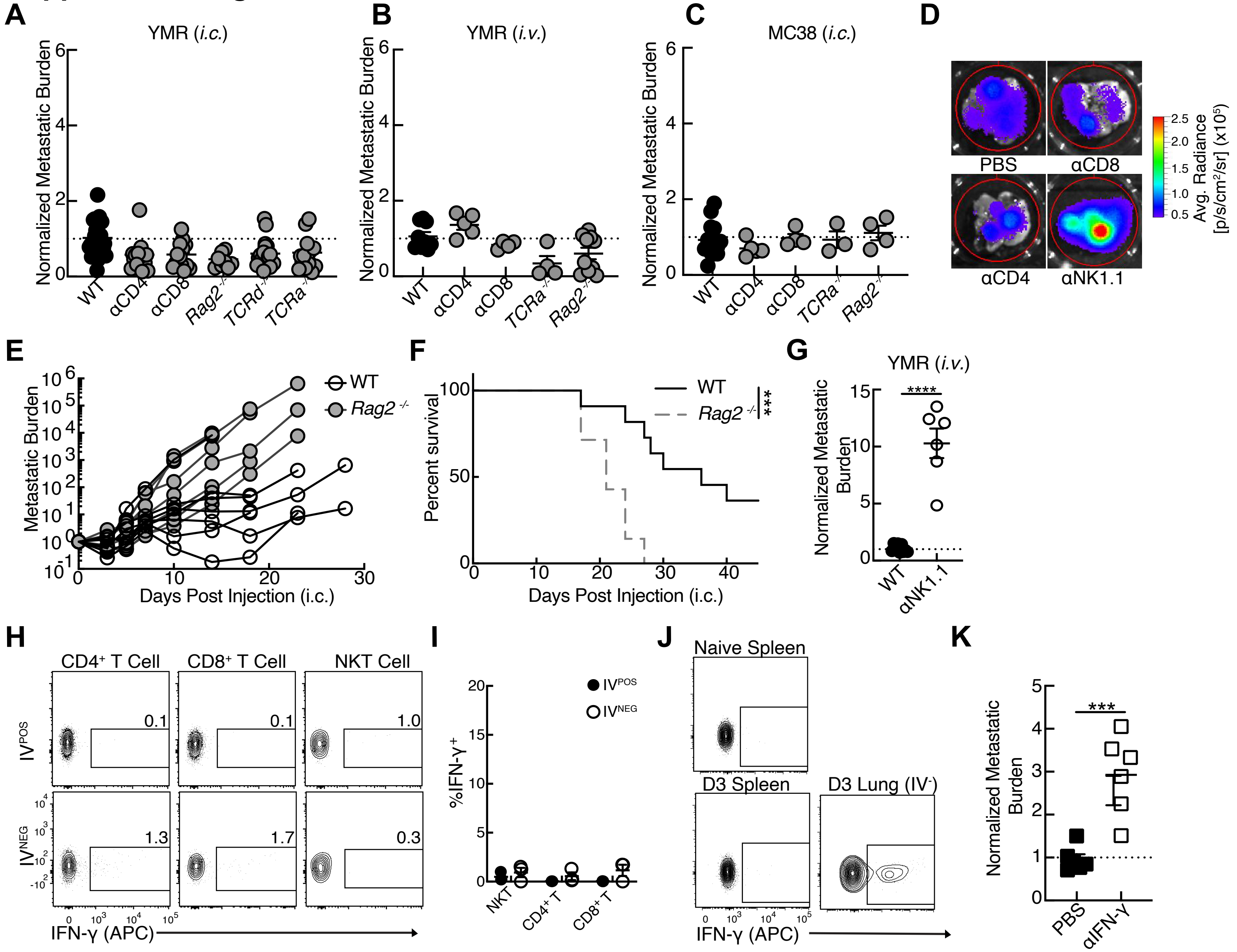

Supplemental Figure 3. **Related to Figure 4. Local NK cells are required to confer initial host anti-metastatic immunity.**
(A-D) Mice were injected with $0.5x10^5$ YMR cells i.c. (A,D), $1x10^5$ YMR cells i.v. (B), and $1x10^5$ MC38 cells i.c.and harvested on day 3 p.i. (A-C) Quantification of normalized metastatic burden in the lung of WT, Rag2$^{-/-}$, Tcra$^{-/-}$,and Tcrd$^{-/-}$ mice, and mice treated with PBS, α-CD4, or α-CD8. (D) Representative image of bioluminescence of whole lung in WT mice treated with PBS, α-CD4, α-CD8, or α-NK1.1 depleting antibody. (E) Longitudinal metastatic growth and (F) Kaplan–Meier survival curves for Rag2$^{-/-}$ and WT mice injected with $0.5x10^5$ YMR-G/L cells i.c. (G) Quantification of normalized metastatic burden in the lung of WT mice treated with NK1.1 depleting antibody and injected with $1x10^5$ YMR i.v. and harvested day 3 p.i. (H-J) WT mice were injected i.c. with $5x10^5$ YMR-GL i.c. and lung and spleen tissues were harvested and analyzed by flow cytometry day 3 p.i. (H) Representative flow plots and (I) quantification and of intracellular IFN-γ staining in indicated CD45.2 IV labeled and unlabeled fraction in the indicated lymphocyte population in the lung. (J) Representative flow plots of intracellular IFN-γ staining in NK cells in the spleen and the lung. (K) Quantification of normalized metastatic burden in the lung of WT mice treated with IFN-γ neutralizing antibody and injected with $1x10^5$ YMR i.v. harvested day 3 p.i. Data is representative of three independent experiments with at least n=5 mice per group. Sample is compared using unpaired Student's t test (G,K) or Gehan-Breslow Wilcoxon test (F). Data is presented as ± SEM. (***$p<0.001$,****$p<0.0001$).

Supplemental Figure 4

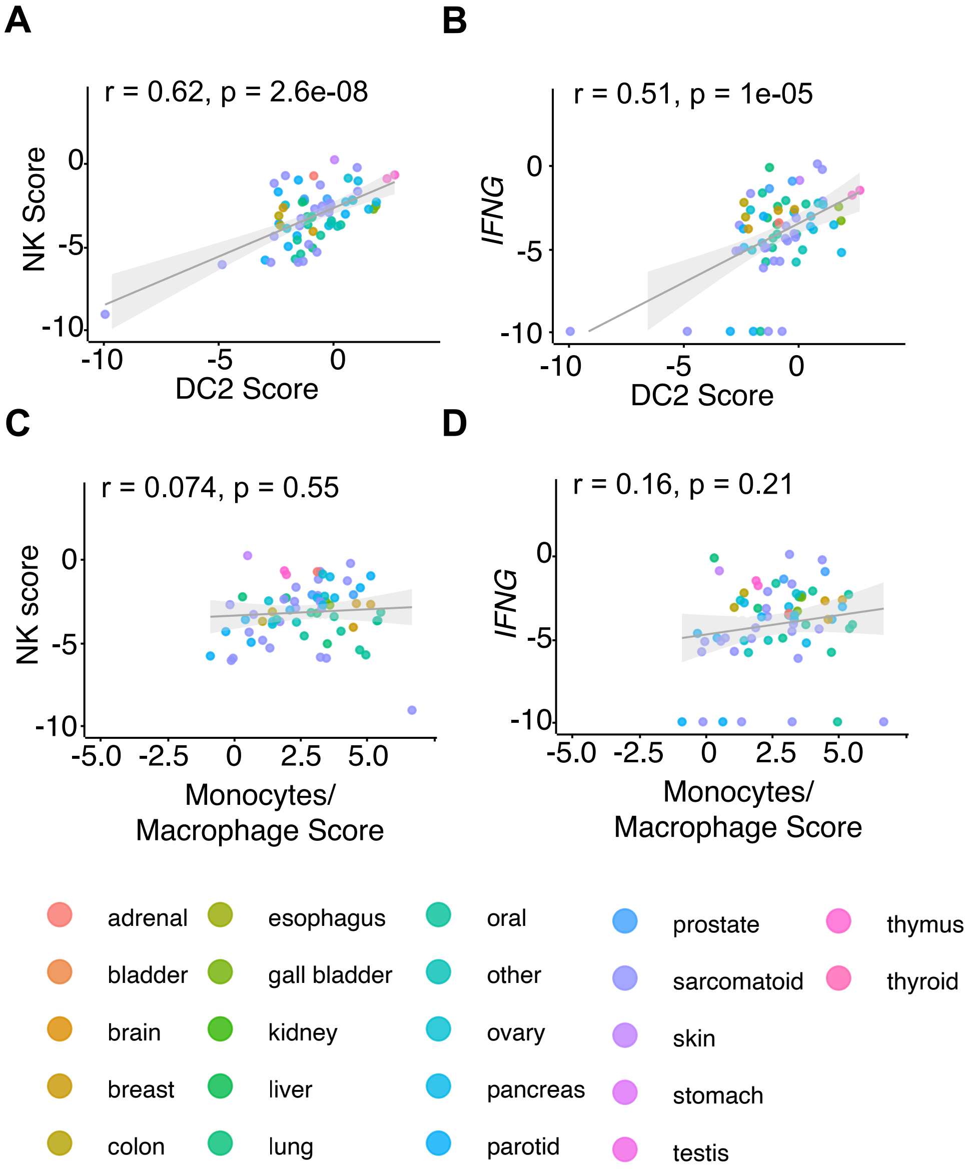

Supplemental Figure 4. Related to Figure 4 and 5. **DC2 signature correlates with NK cell signature and IFNG transcripts in human metastatic data sets.**
(A,B) Pair-wise Pearson's correlation calculated between DC2 signature and NK cell signature (A) or IFNG transcript level (B) in the lungs from the MET500 cohort 38 . (C,D) Pair-wise correlation calculated between monocyte/macrophage signature and NK cell signature (C) or IFNG transcript (D) across in the lung. Each dot represents a distinct metastatic tumor biopsy and is color coded based on primary tumor location (legend below) presented by metastatic location. For each plot, associated Pearson's correlation coefficients and linear regression significance are given. Regression lines are indicated by the gray solid lines. 95% confidence intervals for the regression lines are denoted by the light gray filled areas.
